# Role of Sam68 in Sunitinib induced renal cell carcinoma apoptosis

**DOI:** 10.1002/cam4.4743

**Published:** 2022-04-10

**Authors:** Zeshen Wu, Yulu Peng, Longbin Xiong, Jun Wang, Zhen Li, Kang Ning, Minhua Deng, Ning Wang, Wensu Wei, Zhiyong Li, Pei Dong, Chunping Yu, Fangjian Zhou, Zhiling Zhang

**Affiliations:** ^1^ Department of Urology Sun Yat‐sen University Cancer Center Guangzhou China; ^2^ State Key Laboratory of Oncology in Southern China Guangzhou China; ^3^ Collaborative Innovation Center for Cancer Medicine Guangzhou China

**Keywords:** cell apoptosis, drug sensitivity, renal cell carcinoma, Sam68, sunitinib

## Abstract

Sunitinib is one of the first‐line targeted drugs for metastatic renal cell carcinoma (RCC) with dual effects of antiangiogensis and proapoptosis. Sam68 (Src‐associated in mitosis, 68 KDa), is found being involved in cell apoptosis. This article reveals that Sam68 impacts the sensitivity to sunitinib by mediating the apoptosis of RCC cells. Immunohistochemical staining indicated that the Sam68 expression levels in sunitinib sensitive tumor tissues were markedly higher than those in sunitinib resistant tumor tissues. Sunitinib induced RCC cell apoptosis in a concentration‐dependent manner and inhibited the expression of total and phosphorylated Sam68 (p‐Sam68). Downregulation of Sam68 expression inhibited RCC cell apoptosis induced by sunitinib. While upregulation of Sam68 expression could enhance apoptosis induced by sunitinib. Xenograft models showed that tumors in the Sam68‐knockdown group did not shrink as much as those in the control group after treatment with sunitinib for 4 weeks. Together, our results suggest that Sam68 expression is associated with the sensitivity of ccRCC patients to sunitinib. Sam68 may promote cell apoptosis induced by sunitinib, and the Sam68 expression level may be a biomarker for predicting sunitinib sensitivity in ccRCC patients.

## Graphical abstract

Sam68 expression is associated with the sensitivity to sunitinib in ccRCC patients. Upregulation or downregulation the expression of Sam68 in RCC cells affected apoptosis induced by sunitinib. Sam68 may be a potential target for sunitinib, and may be a biomarker for predicting sunitinib sensitivity in ccRCC patients.

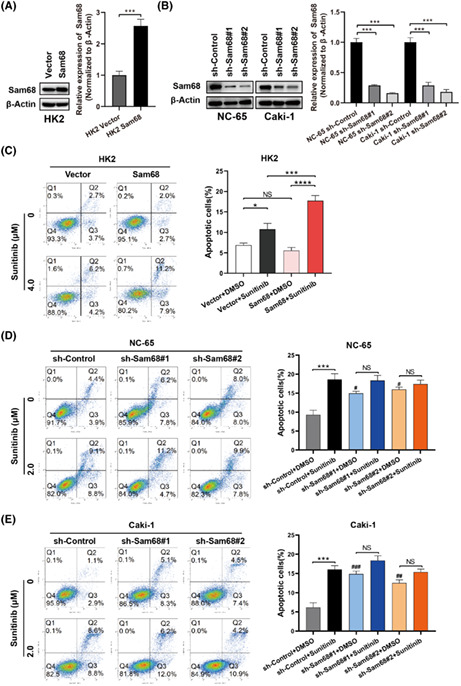

## 1 INTRODUCTION

Kidney cancer is one of the most common neoplasms in the urinary system, and approximately 85% of cases are renal cell carcinoma (RCC).^1^ Studies report that RCC accounts for 5% and 3% of all cancers and ranks as the sixth and ninth most diagnosed cancers in males and females, respectively.^2^, ^3^ The incidence of RCC increased by 0.6% and the death rate fell by 0.7% per year on average from 2006 to 2015, according to the Surveillance, Epidemiology and End Results (SEER) database.^4^ In America, approximately 16% of patients are diagnosed with metastatic disease at the first visit.^2^ For this cohort of patients, systemic therapies, including targeted therapies, immune checkpoint inhibitors, cytoreductive nephrectomy, stereotactic body radiation therapy (SBRT) and ablative techniques, are generally recommended.^5^

As a first‐line targeted drug for relapsed or metastatic RCC, sunitinib exerts anticancer action via its antiangiogenic and proapoptotic effects.^6^, ^7^ Sunitinib is a tyrosine kinase inhibitor (TKI) that has multiple target receptors, including vascular endothelial growth factor receptor 1 (VEGFR‐1), VEGFR‐2, and VEGFR‐3; platelet‐derived growth factor receptor α (PDGFR‐α) and PDGFR‐β; neurotrophic factor receptor (RET); Fms‐like tyrosine kinase 3 (FLT‐3); signal transducer and activator of transcription 3 (Stat3); c‐KIT; and Src.^6^, ^8^, ^9^ Studies have shown that sunitinib inhibits the mitosis of human endothelial cells induced by VEGF and their capacity to form capillaries. The potential antiangiogenic effect allows sunitinib to reduce tumor microvascular density and suppress tumor growth and metastasis in vivo.^10^, ^11^, ^12^ Other studies indicate that sunitinib also causes notable tumor responses directly by inducing tumor cell necrosis, apoptosis or cell cycle arrest in diverse solid neoplasms or tumor cells, such as gastrointestinal stomal tumors (GISTs), neuroblastoma, glioma and urinary bladder‐cancer cells.^7^, ^13^, ^14^, ^15^

In metastatic RCC patients, the objective response rate (ORR) for sunitinib was reported to be 34%–47%.^16^, ^17^, ^18^ However, drug resistance and disease progression occurred after a median of 6–15 months of sunitinib treatment initiation.^19^, ^20^, ^21^ Current studies have identified several possible mechanisms causing sunitinib resistance in RCC, including upregulation of proangiogenic signaling pathways driven by hypoxia, promotion of tumor invasiveness and metastasis by promoting epithelial‐to‐mesenchymal transition (EMT), resistance mediated by the tumor microenvironment via recruitment of bone marrow‐derived cells (BMDCs), activation of alternative signaling pathways promoting proliferation or suppressing apoptosis, inadequate target inhibition by means of single nucleotide polymorphisms (SNPs) or increased lysosomal sequestering, and resistance mediated by the action of microRNAs involved in angiogenesis and apoptosis pathways. On the basis of studies of these mechanisms, a number of potential biomarkers predicting sensitivity or resistance to sunitinib have been found and reported.^20^ However, no markers have been validated that can predict the response to sunitinib therapy in large cohorts of patients and can be used in the clinic. Thus, more investigations are needed to find novel biomarkers for sunitinib sensitivity.

Sam68 (Src‐associated in mitosis, 68 KDa), belonging to the signal transduction and activation of RNA metabolism (STAR) family,^22^, ^23^ was originally discovered as a Src‐associated substrate in cell mitosis.^24^, ^25^ In recent decades, Sam68 has been reported to participate in a series of cellular processes, including transcription,^22^, ^26^ translation,^27^ signal transduction,^28^, ^29^ RNA splicing and export,^29^, ^30^, ^31^ cell cycle progression and apoptosis,^32^, ^33^, ^34^ and replication of certain viruses.^35^, ^36^ As one of the RNA‐binding proteins (RBPs), the posttranslational modifications of Sam68 impact the subcellular localization and affinity for RNA, such as phosphorylation, methylation, acetylation, and small ubiquitin‐like modifier (SUMO).^34^, ^37^, ^38^, ^39^ Sam68 mainly localizes in the nucleus via its RNA‐binding domain, which has nonconventional nuclear localization signals, and plays a role in pre‐mRNA processing.^25^, ^40^ When tyrosine is phosphorylated by Src‐like kinase, the RNA binding affinity of Sam68 decreases, and it relocalizes to the cytoplasm.^41^ Several studies have revealed that Sam68 is upregulated and functions as an oncogene in many human cancers, such as breast, prostate, cervical cancers and non‐Hodgkin's lymphoma (NHL).^42^, ^43^, ^44^, ^45^

Our previous study found that Sam68 was obviously overexpressed in both RCC tissues and cell lines. High expression and cytoplasmic localization of Sam68 were significantly correlated with poor overall survival in patients with RCC.^46^ In this study, we report that Sam68 plays an essential role in the apoptotic effect of sunitinib on renal cell carcinoma.

## 2 MATERIALS AND METHODS

### 2.1 Patient information and tissue samples

Forty‐seven patients with relapsed/metastatic RCC who underwent operations or biopsies and were histopathologically diagnosed, were then treated with sunitinib at Sun Yat‐sen University Cancer Center from 2010 to 2018. According to the Response Evaluation Criteria In Solid Tumors (RECIST 1.1), patients with evaluation of Complete Response (CR) and Partial Response (PR) were classified into the sunitinib sensitive group, while patients with evaluation of Stable Disease (SD) and Progress Disease (PD) were classified into the sunitinib resistant group. All patients signed an informed consent form before the study. Paraffin‐embedded samples of patient tumors and the clinical information of patients were obtained for research purposes with the approval of the Institutional Research Ethics Committee of Sun Yat‐sen University Cancer Center.

### 2.2 Immunohistochemical analysis

Immunohistochemistry (IHC) was employed to detect Sam68 protein expression in 47 human RCC tissues with the human Sam68 antibody (1:300, Abcam, Cambridge, England). The immunohistochemistry procedure was performed according to the manufacturer's instructions. The immunostaining intensity of the tissue sections was measured by FIJI software.^47^ Optical density (OD) was calculated using the following formula: OD = log(max intensity/mean intensity) × 100. The OD value was positively correlated with the protein expression level of Sam68.

### 2.3 Cell lines

The human normal renal tubular epithelial cell line HK2 was cultured in DMEM/F12 (Invitrogen, California, America) supplemented with 10% fetal bovine serum (Gibco, California, America). The human embryonic kidney cell line 293 T and RCC cell lines 769P, NC‐65, ACHN, SKRC39, and UMRC6 were cultured in DMEM (Invitrogen, California, America), while Caki‐1 and 786O cells were cultured in RPMI 1640 (Invitrogen, California, America) supplemented with 10% fetal bovine serum. Cells were obtained from the American Type Culture Collection (ATCC, Manassas, America) and incubated at 37°C and 5% CO_2_.

### 2.4 Plasmids and retroviral infection

The PCR‐amplified human Sam68 coding sequence was subcloned into the pBABE retroviral vector and then the Sam68 overexpression plasmid was constructed. Sam68 short hairpin RNA (shRNA) oligonucleotides (shRNA#1: 5′‐GGACCACAAGGGAATACAATC‐3′; shRNA#2: 5′‐GCATCCAGAGGATACCTTTGC‐3′) were designed on the website (http://t.cn/S44oNe; created by Sigma‐Aldrich, Darmstadt, Germany), synthesized (RuiBiotech, Beijing, China), and then subcloned into pLKO.1 carriers to construct Sam68 knockdown plasmids. Transfections were performed with Lipofectamine 3000 (Invitrogen, California, America) according to the manufacturer's instructions. Stably transfected cell lines were selected with 0.5 μg/mL puromycin for more than 10 days.

### 2.5 RNA extraction and reverse transcription and quantitative real‐time polymerase chain reaction (qRT‐PCR)

Total RNA was extracted from cell lines using TRIzol reagent (Invitrogen, California, America), and 2 μg of RNA from each sample was acquired for reverse transcription. Then, the products were used for cDNA synthesis according to the manufacturer's instructions, and qRT‐PCR was adopted to assess the fold changes in Sam68 mRNA expression. The mRNA expression of the housekeeping gene beta actin (β‐Actin) was applied to normalize the geometric mean for each sample. Primers for Sam68 and β‐Actin were generated and verified using the Primer‐BLAST modules on the National Center for Biotechnology Information (NCBI) website (https://www.ncbi.nlm.nih.gov/tools/primer‐blast/). The primer sequences were as follows: Sam68 forward: 5′‐AAAGAGCGAGTGCTGATACCT‐3′, reverse: 5′‐TGAGCCCTTTCCCA ATACAGA‐3′; β‐Actin forward: 5′‐CTTCGCGGGCGACGAT‐3′, reverse: 5′‐CCACATAGG AATCCTTCTGACC‐3′.

### 2.6 Western blotting

Total protein was extracted from floating and attached cells using RIPA lysis buffer (GenStar, Beijing, China). A bicinchoninic acid (BCA) assay kit (GenStar, Beijing, China) was applied for protein quantification following the manufacturer's instructions. Western blotting was carried out according to standard methods described in the manufacturer's protocol. In brief, equal amounts of proteins mixed with 5× sodium dodecyl sulfate‐polyacrylamide gel electrophoresis (SDS‐PAGE) loading buffer (GenStar, Beijing, China) were heated in a metal bath at 100°C for 10 min, electrophoresed through 10% SDS‐PAGE gels and electrotransferred onto polyvinylidene fluoride (PVDF) membranes (Merck Millipore, Darmstadt, Germany). After the membranes were blocked in blocking solution containing 5% skim milk for 1 h at room temperature, they were incubated with certain primary antibodies (Sam68: 1:500, Abcam, Cambridge, England; p‐Sam68: 1:500, Kerafast, Boston, America; Src, p‐Src, cleaved‐PARP and cleaved‐Caspase3: 1:500, CST, Boston, America; Bcl‐x: 1:500, BD Biosciences, New Jersey, America; β‐Actin: 1:1000, Sigma, Darmstadt, Germany) overnight at 4°C, and then incubated with corresponding secondary antibodies (with rabbit or mouse reactivity; 1:5000, Abcam, Cambridge, England) for 1 h at room temperature. The protein bands were visualized using an enhanced chemiluminescence (ECL) substrate (Tanon, Shanghai, China). Anti‐β‐Actin antibody was used as the loading control. The intensities of bands were detected and quantified by ImageJ software.

### 2.7 Cell Counting Kit‐8 (CCK‐8) assay

Cells were seeded into 96‐well plates at 2000 cells/well and allowed to attach overnight. Vehicle dimethyl sulfoxide (DMSO; Merck Millipore, Darmstadt, Germany) or sunitinib (Selleck, Shanghai, China; dissolved in DMSO) was added to the medium at different concentrations for the following days. After 24, 48 or 72 h, CCK‐8 reagents (APExBio, Houston, America) were added to each well and then incubated at 37°C for 2 h. Absorbances at 450 nm were read using an automated plate reader. The half maximal inhibitory concentrations (IC_50_) of sunitinib treatment for 48 h in HK2, NC‐65 Caki‐1, 769P and SKRC39 cells were then calculated.

### 2.8 Apoptosis assays

To analyze cell apoptosis levels, cells were seeded into 6‐well plates at 1 × 10^5^ cells/well. After overnight attachment, DMSO or sunitinib was added to treat the cells for 48 h. Then, floating and attached cells were collected and stained with allophycocyanin (Annexin V‐APC; KeyGEN, Jiangsu, China) and 4′, 6‐diamidino‐2‐phenylindole (DAPI; KeyGEN, Jiangsu, China). Apoptosis rates were measured by flow cytometry and analyzed by FlowJo software.

### 2.9 Xenograft mouse model

Six‐week‐old female BALB/c nude mice were obtained from Beijing Vital River Laboratory Animal Technology Company (Beijing, China), randomized into two groups and anesthetized. NC‐65 cells (5 × 10^6^) transfected with control shRNA (sh‐Control) or Sam68 shRNA (sh‐Sam68) were subcutaneously injected into the rear back region of each mouse. When tumors reached 70‐100 mm^3^ at an average of 28 days later, each group of mice was randomized into two subgroups (*n* = 5 each) and treated with 0.5% carboxymethylcellulose sodium (CMC‐Na; Selleck, Shanghai, China; as placebo) or sunitinib (suspended in 0.5% CMC‐Na; 40 mg/kg/day) by gavage. Tumor volume was measured every 2–3 days and calculated as follows: volume = length×(width)^2^/2. The mice were euthanized 4 weeks after sunitinib treatment, and then tumors were resected and subsequently weighed. All animal experimental procedures were approved by the Laboratory Animal Ethics Committee of Sun Yat‐sen University Cancer Center.

### 2.10 Statistical analysis

Every experiment was performed at least 3 times. All statistical analyses were calculated from at least three independent experiments using GraphPad Prism 8.0 and SPSS 25.0 software. Two‐tailed Student's *t*‐test or Chi‐square test was used to measure differences between groups and the Log‐rank test was adopted to evaluate the PFS between the two groups. Differences with *p* < 0.05 were considered statistically significant in all cases.

## 3 RESULTS

### 3.1 The Sam68 expression level is correlated with the sensitivity to sunitinib in ccRCC

**FIGURE 1:**
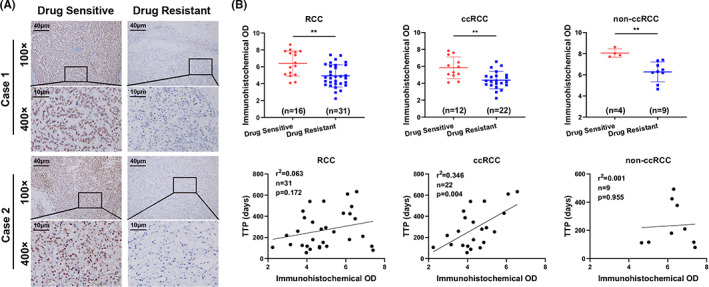
Sam68 expression correlates with sunitinib drug sensitivity in renal cell carcinoma (RCC). (A) Immunohistochemistry (IHC) showed that Sam68 expression was markedly higher in sunitinib sensitive tumor samples than in sunitinib resistant tumor samples. (B) Top: The average immunohistochemical optical density (OD) calculated by FIJI software in sunitinib drug sensitive tumors was significantly higher than that in sunitinib resistant tumors; Bottom: The immunohistochemical OD was positively correlated with the time to progression (TTP) in the clear cell renal cell carcinoma (ccRCC) subgroup but not in the sunitinib resistant RCC cohort or the non‐clear cell renal cell carcinoma (non‐ccRCC) subgroup. **: *p* < 0.01

To determine the relationship between the levels of Sam68 expression and the sensitivity of RCC to sunitinib, an IHC assay was employed. Forty‐seven paraffin‐embedded tumor tissue samples of relapsed or metastatic RCC, including 16 sunitinib sensitive and 31 sunitinib resistant patients, were immunohistochemically stained. The IHC results revealed that the average Sam68 expression in sunitinib sensitive tumors was significantly higher than that in sunitinib resistant tumors (Figure 1A), and statistical analyses showed that the average immunohistochemical OD in sunitinib drug sensitive tumors was significantly higher than that in sunitinib resistant tumors (6.393 vs. 4.927, *p* = 0.001; Table 1, Figure 1B, top). However, the drug response to sunitinib had no correlation with age, sex, pathologic stage or pathologic diagnosis (*p* > 0.05; Table 1).

**TABLE 1:** Relationship between sunitinib drug response and clinicopathological features of 47 relapsed /metastatic RCC patients

Variables	Total	Sunitinib sensitive	Sunitinib resistant	*p* value
Patients (*n*)	47	16 (34.0%)	31(66.0%)	
Age (years)				
≤56	22	8 (36.4%)	14 (63.6%)	0.755
>56	25	8 (32.0%)	17 (68.0%)	
Gender				
Male	32	11 (34.4%)	21 (65.6%)	0.945
Female	15	5 (33.3%)	10 (66.7%)	
Pathologic diagnosis				
ccRCC	34	12 (35.3%)	22 (64.7%)	0.772
non‐ccRCC	13	4 (30.8%)	9 (69.2%)	
Pathologic stage				
III	10	1 (10.0%)	9 (90.0%)	0.074
IV	37	15 (40.5%)	22 (59.5%)	
Relasped/Metastatic lesion				
Unifocal	25	10 (40.0%)	15 (60.0%)	0.363
Multifocal	22	6 (27.3%)	16 (72.7%)	
Immunohistochemical OD (Sam68)	5.426 ± 1.536	6.393 ± 1.493	4.927 ± 1.321	**0.001**
Subcellular localization				
Nucleus	41	15 (36.6%)	26 (63.4%)	0.341
Cytoplasm	6	1 (16.7%)	5 (83.3%)	
PFS (days)	377 (153–699)	966 (687–1409)	219 (116–424)	**<0.001**

*Note*: Differences between groups were measured by Two‐tailed Student's *t*‐test or Chi‐square test and the PFS was evaluated by Log‐rank test. Scale variables were expressed as mean ± standard deviation or median (interquartile range); ordinal and nominal variables were expressed as number or number (percentages). *p* value < 0.05 was considered significant (in bold).

In addition, Sam68 was highly expressed in the sunitinib sensitive group and was mostly localized in the nucleus (15/16; Table 1, Figure 1A), and the median progression free survival (PFS) of sunitinib sensitive patients was markedly longer than that of sunitinib resistant patients (966 vs. 219 days, *p* < 0.001; Table 1). The two groups of patients were then divided into two subgroups, namely, the clear cell renal cell carcinoma (ccRCC) group and the non‐clear cell renal cell carcinoma (non‐ccRCC) group, according to their pathologic diagnosis, and the immunohistochemical OD value analysis results were the same as those previously mentioned (Figure 1B, top).

The correlations between the time to progression (TTP) of patients after sunitinib treatment initiation and Sam68 immunohistochemical OD were then investigated in the sunitinib resistant cohort and the two corresponding subgroups. TTP was positively correlated with the Sam68 immunohistochemical OD values in the ccRCC subgroup (*p* = 0.004) but not in the sunitinib resistant RCC and non‐ccRCC subgroup (*p* > 0.05, Figure 1B, bottom).

These results suggested that the expression of Sam68 in sunitinib sensitive tumors was higher than that in sunitinib resistant tumors in both ccRCC and non‐ccRCC subgroups, and had a positive correlation with TTP in ccRCC patients.

### 3.2 Sunitinib inhibits the phosphorylation of Sam68

**FIGURE 2:**
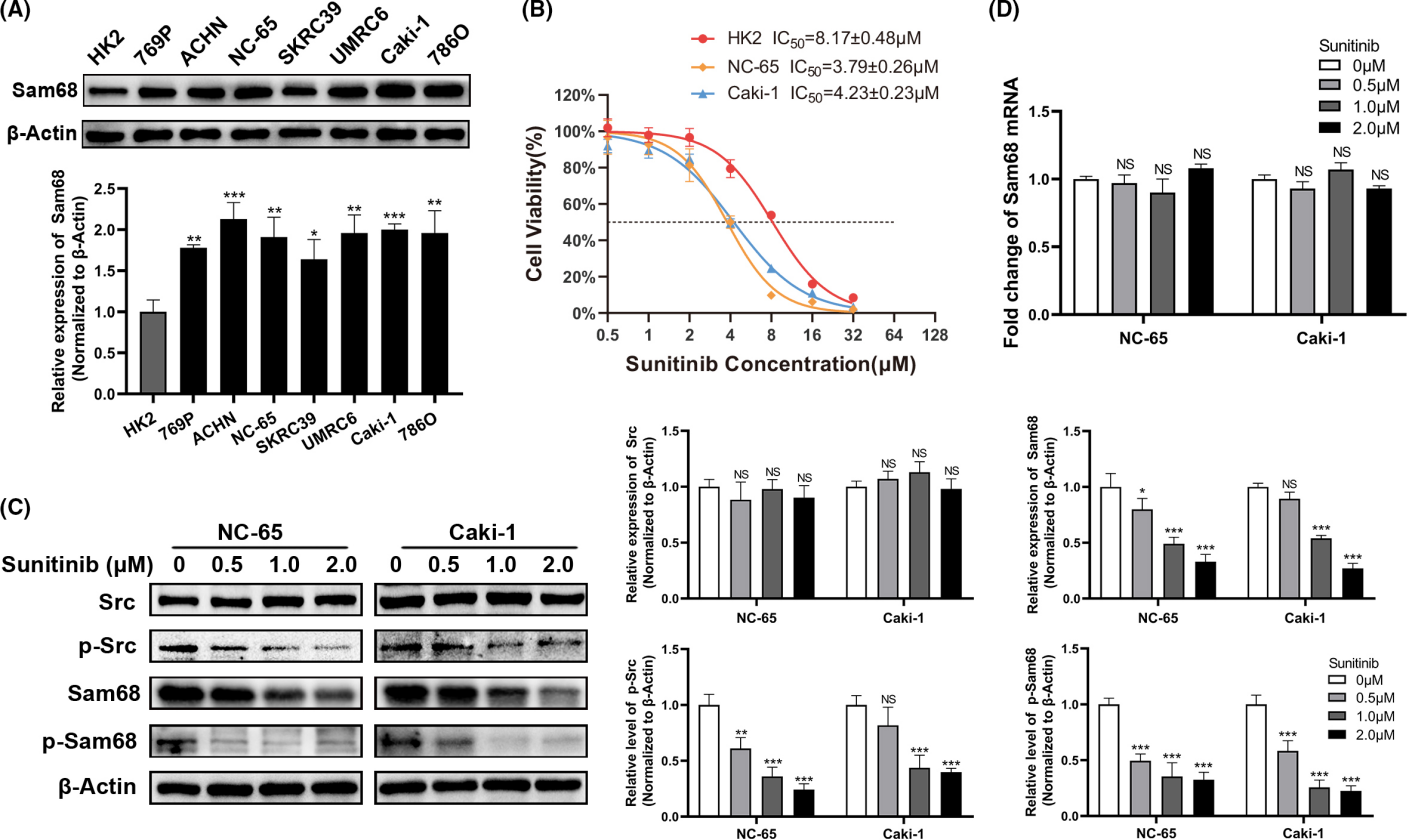
Sunitinib inhibits the total expression and phosphorylation levels of Sam68. (A) Western blotting showed that Sam68 expression in RCC cell lines was significantly higher than that in HK2 cells. (B) The dose–response curves and the corresponding half maximal inhibitory concentrations (IC_50_) of sunitinib in HK2, NC‐65 and Caki‐1 cells after treatment for 48 h were shown. (C) The total protein level of Src did not significantly change but the phosphorylation levels of Src (p‐Src) apparently decreased with increasing doses of sunitinib, while the total expression and phosphorylation levels of Sam68 were both markedly decreased. (D) The qRT‐PCR results showed that the transcript levels of Sam68 were not affected by sunitinib treatment. NS: no significant; *: *p* < 0.05; **: *p* < 0.01; ***: *p* < 0.001

To determine the appropriate cell lines for further experiments, the Sam68 expression levels were re‐examined in RCC cell lines and HK2 cells. Western blotting showed that the expression of Sam68 was clearly higher in the RCC cell lines than in the HK2 cells (Figure 2A). NC‐65, Caki‐1, HK2, 769P and SKRC39 cell lines were chosen for further experiments and the IC_50_ values of sunitinib treatment for 48 h were 3.79 μM, 4.23 μM, 8.17 μM, 3.57 μM and 4.58 μM, respectively (Figure 2B; Figure S1A).

NC‐65 and Caki‐1 cells were treated with a concentration gradient of sunitinib for 48 h, and the total RNAs and proteins were extracted. The total expression and phosphorylation level of Src were examined. The level of phosphorylated Src (p‐Src) was decreased gradually in both NC‐65 and Caki‐1 cells treated with an increasing dose of sunitinib, while the total Src protein levels were hardly affected (Figure 2C). This result demonstrated that sunitinib obviously inhibited the activity of Src in RCC cells.

As Sam68 is a substrate of Src, we further assessed the potential effects of sunitinib on Sam68. Western blotting showed that total Sam68 protein and phosphorylated Sam68 (p‐Sam68) levels decreased in a dose dependent manner with sunitinib (Figure 2C). Furthermore, qRT‐PCR was performed to detect the influence of sunitinib on Sam68 at the transcriptional level, and the data suggested no significant changes in Sam68 mRNA despite an increasing dose of sunitinib (Figure 2D).

These results suggested that sunitinib inhibits the expression and activity of Sam68. Sam68 might be a potential target of sunitinib, and play a crucial role in the effect induced by sunitinib in RCC cells.

### 3.3 Sam68 increases the efficiency of sunitinib‐induced cell apoptosis in RCC

**FIGURE 3:**
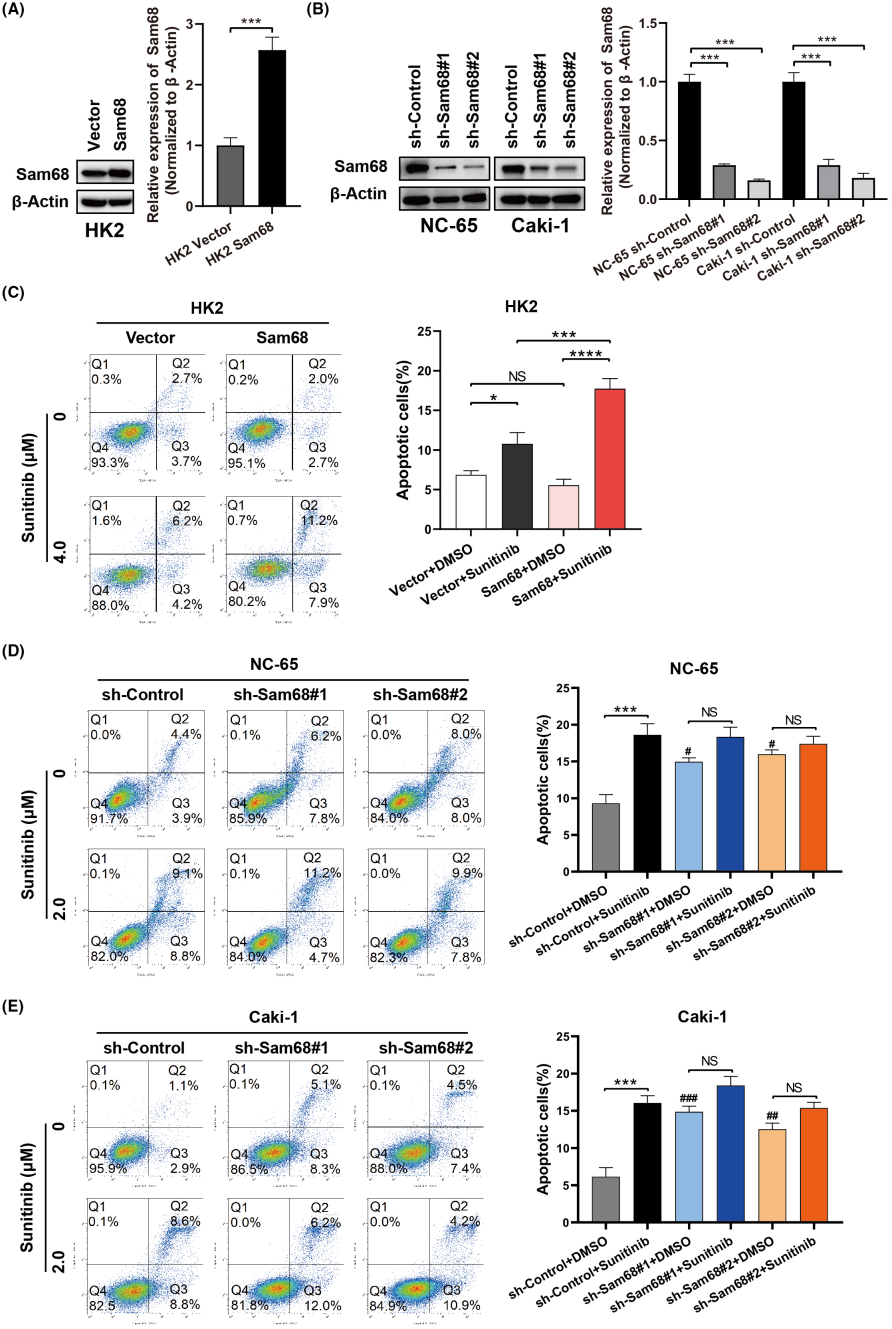
Sam68 increases RCC cell apoptosis induced by sunitinib. (A,B) Western blotting verified the efficiency of Sam68 upregulation in HK2 cells (A) and downregulation in NC‐65 and Caki‐1 cells (B). (C–E) Flow cytometry plots showed that the apoptosis rates induced by sunitinib were obviously increased in Sam68 overexpressing HK2 cells compared to the vector control cells (C, left); the apoptosis rate in Sam68‐knockdown cells did not increase significantly after sunitinib treatment (D,E, left); the corresponding proportions of apoptotic cells were shown in the bar graphs (right). NS: no significant; *: *p* < 0.05; **: *p* < 0.01; ***: *p* < 0.001; ****: *p* < 0.0001; #: *p* < 0.05 versus sh‐Control+DMSO group; ##: *p* < 0.01 versus sh‐Control+DMSO group; ###: *p* < 0.001 versus sh‐Control+DMSO group

To further confirm the impact of Sam68 in the sunitinib treatment of RCC, HK2, 769P and SKRC39 cells were used to constructed exogenous Sam68‐overexpression cell line while Caki‐1 and NC‐65 cells were selected to established Sam68‐knockdown cell lines (Figure 3A,B; Figure S1B). Stably transfected cells were treated with the proper concentration of sunitinib or DMSO for 48 h, and the apoptotic cell rates were detected by flow cytometry. The apoptosis rate of the Sam68‐overexpressing HK2 cells was obviously increased after sunitinib treatment, compared to that of the vector control cells (Figure 3C), and the same results were shown in both 769P and SKRC39 cells (Figure S1C,D). In the Sam68‐knockdown cell lines, overlooking the apoptosis effects resulting from the downregulation of Sam68 gene expression, the apoptosis rates caused by sunitinib treatment were not markedly increased, compared with those of the control cells (Figure 3D,E). These results indicated that Sam68 increased the efficiency of cell apoptosis induced by sunitinib in RCC.

### 3.4 Downregulation of Sam68 weakens the antitumor effect of sunitinib in vitro and in vivo

**FIGURE 4:**
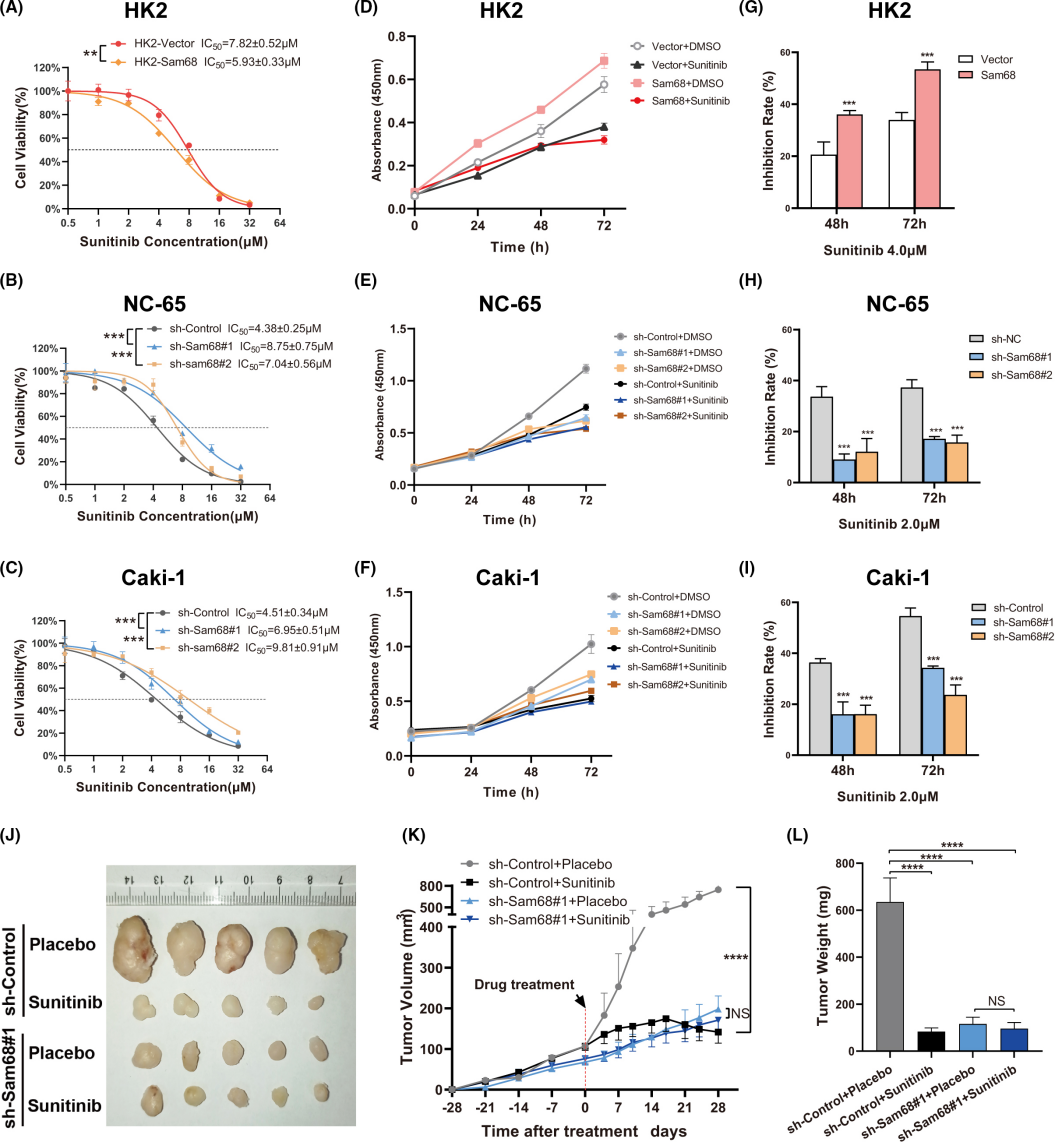
Downregulation of Sam68 weakens the antitumor effect of sunitinib in vitro and in vivo. (A–C) The dose–response curves and the corresponding half maximal inhibitory concentrations (IC_50_) of sunitinib were changed in HK2, NC‐65 and Caki‐1 cells while Sam68 were upregulated or downregulated. (D–F) The growth curves showed that the growth rates were significantly suppressed in Sam68 overexpressing HK2 cells (D) and were not obviously suppressed in Sam68‐knockdown NC‐65 and Caki‐1 cells (E,F). (G–I) The bar graphs showed that the growth inhibition rates in cells were changed after sunitinib treatment for 48 and 72 h when Sam68 expression was upregulated or downregulated. (J–L) The tumor volume (J,K) and weight (L) were obviously reduced and the growth rate of tumors was obviously suppressed after sunitinib treatment in the control group but not in Sam68 downregulation group. NS: no significant; **: *p* < 0.01; ***: *p* < 0.001; ****: *p* < 0.0001

In vitro, the CCK‐8 assay indicated that the IC_50_ values of sunitinib treatment for 48 h were significantly decreased in HK2, 769P and SKRC39 cells while Sam68 expressions were upregulated, and notably increased in NC‐65 or Caki‐1 Sam68‐knockdown cells (Figure 4A–C; Figure S2A,B). Cell growth was further suppressed in HK2, 769P and SKRC39 cells when Sam68 expression was upregulated and was not obviously suppressed in NC‐65 or Caki‐1 Sam68‐knockdown cells, after treatment with sunitinib for a few days (Figure 4D–F; Figure S2C,D). The inhibition rates after sunitinib treatment for 48 and 72 h were calculated and shown to further clarify these consequences (Figure 4G–I; Figure S2E,F).

In vivo, xenograft models showed that both tumor volume (Figure 4J,K) and weight (Figure 4L) were obviously reduced after sunitinib treatment for 4 weeks in the control group, while the tumor volume and weight were reduced slightly because of a reduction in Sam68 expression in tumor cells.

These results showed that downregulation of Sam68 reduces the drug sensitivity of sunitinib, weakens the tumor growth inhibition effect of sunitinib by inhibiting cell apoptosis both in vitro and in vivo.

### 3.5 Sam68 mediates the sunitinib apoptotic effect by modulating the alternative splicing of Bcl‐x

**FIGURE 5:**
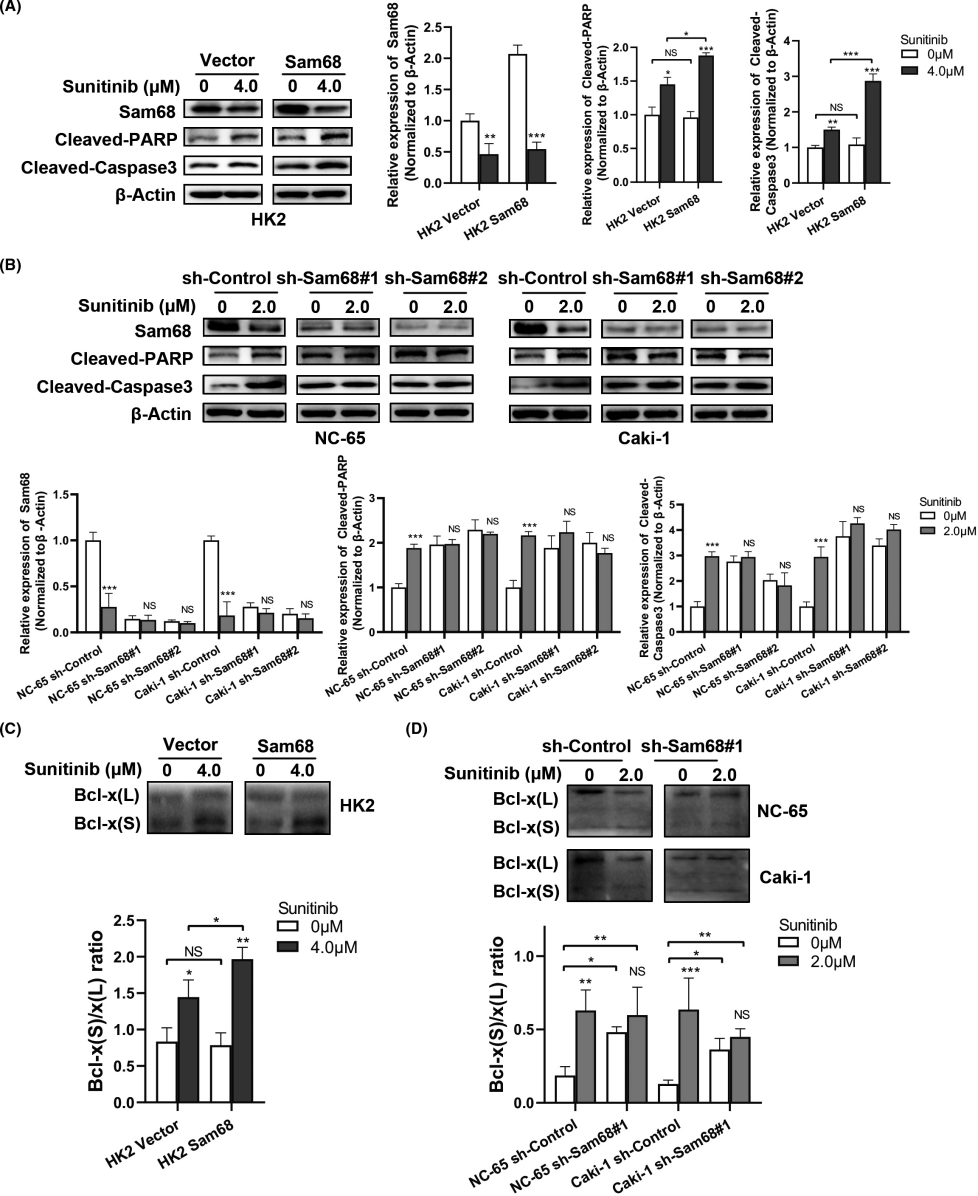
Sam68 mediates sunitinib apoptotic effects by modulating the alternative splicing of Bcl‐x. (A,B) Western blotting showed changes in cleaved‐PARP and cleaved‐Caspase3 levels with or without sunitinib treatment in RCC cells after upregulating or downregulating the expression of Sam68. (C,D) The alternative splicing of Bcl‐x(S) increased obviously after sunitinib treatment in Sam68 upregulated cells (C), while the expression of Bcl‐x(L) or Bcl‐x(S) changed observably in Sam68 downregulated cells (D); the bar graphs showed the corresponding changes in Bcl‐x(S)/Bcl‐x(L) ratios (right). NS: no significant; *: *p* < 0.05; **: *p* < 0.01; ***: *p* < 0.001

Changes in cleaved PARP and cleaved caspase3 levels before and after sunitinib treatment were detected, according to the previous results. Western blotting showed that the protein expression of cleaved PARP and cleaved caspase3 was significantly increased when Sam68 expression was upregulated, but not obviously increased when Sam68 was downregulated (Figure 5A,B).

Changes in Bcl‐x alternative splicing were also detected because previous studies reported that Sam68 mediates cell apoptosis by modulating the alternative splicing of Bcl‐x.^30^, ^33^ Western blotting showed that the alternative splicing of Bcl‐x(L) and Bcl‐x(S) was changed after sunitinib treatment, which exerted antiapoptotic and proapoptotic effects, respectively. The alternative splicing of Bcl‐x(S) was further increased after sunitinib treatment in HK2 cells overexpressing Sam68. However, sunitinib treatment neither increased the expression of Bcl‐x(S) nor observably decreased the expression of Bcl‐x(L), while the Sam68 expression was obviously inhibited in RCC cells. The ratios of Bcl‐x(S)/Bcl‐x(L) were determined, and the results showed that Bcl‐x(S)/Bcl‐x(L) ratio was apparently increased in Sam68‐overexpression cells but slightly increased in Sam68‐knockdown cells, after sunitinib treatment (Figure 5C,D).

These results suggested that Sam68 might mediate cell apoptosis caused by sunitinib via modulating the alternative splicing of Bcl‐x.

## 4 DISCUSSION

In the present study, we reported the effect of the Sam68 expression level on sunitinib drug sensitivity in RCC and the potential underlying molecular mechanism. We firstly found that Sam68 expression levels in sunitinib sensitive RCC tumor tissues were higher than in sunitinib resistant ones, and the Sam68 expression level was correlated with sunitinib sensitivity in ccRCC patients. We also found that sunitinib inhibits the total expression and phosphorylation of Sam68. Upregulation of Sam68 enhanced the cell apoptotic effect of sunitinib, while downregulation of Sam68 expression inhibited the sunitinib‐induced apoptosis. Subsequently, the in vitro and in vivo experiments showed that the antitumor effect of sunitinib was significantly affected by the expression of Sam68. Then we preliminary detected that Sam68 may interfere the sunitinib effect via modulating the alternative splicing of Bcl‐x.

RCC is one of the most common and lethal urologic cancers. Quite a few patients have metastasis at the first visit or eventually develop metastatic disease, which indicates a poor prognosis.^2^, ^3^, ^20^ Sunitinib, the most widely used first‐line targeted drug as well as pazopanib, has been reported in several clinical studies with satisfying objective response rates and clinical outcomes in relapsed or stage IV RCC patients at initial treatment.^17^, ^18^, ^19^, ^48^ Frustratingly, disease progression ultimately occurs because of intrinsic or acquired drug resistance. Current studies have reported some possible theories to explain sunitinib resistance. However, its exact molecular mechanisms are still unclear, and explorative studies have continued. Xin Hong et al.^6^ reported that Stat3 inhibition might mediate the immunomodulatory effects of sunitinib and permit the direct proapoptotic effects of sunitinib in RCC cells. Stat3 inhibition also positively affects the tumor immunologic microenvironment by reducing the proportion of immunosuppressive cells. Huang Hai et al.^49^ reported that eukaryotic initiation factor 3 subunit d (EIF3D) could interact with glucose regulated protein 78 (GRP78) and inhibit its degradation, further activating the unfolded protein response (UPR) signaling pathways and maintaining endoplasmic reticulum stress (ERS) homeostasis, thus promoting sunitinib resistance in RCC. Moreover, S.C. Joosten et al.^20^ summarized several mechanisms of sunitinib resistance in RCC patients and identified some potential predictive biomarkers of sensitivity or resistance to sunitinib, such as VEGF, VEGFR, interleukin‐8 (IL‐8), neutrophil gelatinase‐associated lipocalin (NGAL), tumor necrosis factor‐alpha (TNF‐α) and matrix metalloproteinase‐9 (MMP‐9), on the basis of data from current preclinical and clinical studies. However, none of these biomarkers has been validated and widely used in the clinic until now. Therefore, biomarkers that can accurately identify patients who may potentially be resistant to sunitinib are needed to help better manage patients with metastatic RCC.

The function of Sam68 in tumor progression is complicated and multifaceted, due to its participation in a series of cellular processes. Some studies have indicated that Sam68 plays a role as a tumor suppressor gene. Taylor Stephen J et al. ^32^ reported that overexpression of Sam68 results in cell apoptosis or cell cycle arrest in mouse fibroblasts and that trichostatin A (TSA) enhances Sam68‐induced apoptosis. K Liu et al.^50^ reported that Sam68 deficiency leads to neoplastic transformation and tumorigenesis in murine NIH3T3 fibroblasts. However, recent studies have demonstrated that Sam68 acts as an oncogene in many human cancers. Song L et al.^42^ reported that upregulation of Sam68 and its cytoplasmic localization are correlated with a poor outcome in breast cancer, and that downregulation of Sam68 suppresses cell proliferation and tumorigenicity by arresting cells at the G1 to S phase transition. Li Z et al.^44^ also found that Sam68 impacts cellular motility and invasion in cervical cancer, and that its high expression level and cytoplasmic localization were associated with pelvic lymph node metastasis and could be independent prognostic factors for predicting the OS time and the disease free survival (DFS) time. In prostate cancer, Busà R et al.^43^ reported that downregulation of Sam68 delays cell cycle progression, and sensitizes cells to apoptosis induced by DNA damaging agents. Wu Y et al.^45^ reported that Sam68 promotes cell proliferation and inhibits cell apoptosis regulated cell adhesion‐mediated drug resistance (CAM‐DR) via the AKT pathway in NHL. These studies suggest that Sam68 may play diverse roles in different cancers, and even exert inverse effects with different subcellular localizations.

In our previous study, we demonstrated that Sam68 expression and localization were correlated with the clinicopathologic grading of human renal carcinoma. High expression levels of Sam68 might serve as a prognostic factor for RCC patients and its cytoplasmic localization could be an independent predictor of poor prognosis.^46^ However, the molecular mechanisms by which cytoplasmic Sam68 is involved in RCC tumorigenesis and progression are still undiscovered. Nuclear Sam68 is reported to bind with the mRNA of Bcl‐x and modulate its alternative splicing.^30^ Bcl‐x is one of the classical and crucial genes that regulates cell apoptosis, which can alternatively splice into proapoptotic Bcl‐x(S) and antiapoptotic Bcl‐x(L).

In our current study, the Sam68 expression level was found to be associated with sunitinib sensitivity in ccRCC patients. Experiment results, especially xenograft models, strongly demonstrated that Sam68 expression affected the antitumor effect of sunitinib. Therefore, we speculate that Sam68 may be a novel potential target of sunitinib in RCC, especially in ccRCC cells. We also tried to explain the possibly mechanism of Sam68‐mediated apoptosis. Sunitinib inhibited the total expression and phosphorylation of Sam68. It might result in the instability of Sam68, impacting its capacity to modulate Bcl‐x mRNA alternative splicing. High expression levels of Sam68 imply that sunitinib may cause a high production of Bcl‐x(S) and contribute to a high rate of cell apoptosis. When Sam68 was downregulated, the impact of sunitinib on Bcl‐x alternative splicing was reduced. Bcl‐x(S) was not increased and Bcl‐x(L) was not obviously decreased. As a result, cell apoptosis induced by sunitinib was attenuated. On the basis of these results, we reveal that the apoptotic effect induced by sunitinib in RCC tumor cells, at least in part, is regulated by nuclear Sam68 mediated modulation of the alternative splicing of Bcl‐x. These could be critical factors to consider, when selecting the best targeted drugs for RCC patients who accept sunitinib treatment. However, these findings still require further clinical investigation in a larger cohort to confirm their accuracy and practicability and further experiments are needed to verify the molecular mechanism.

In summary, our study demonstrated that Sam68 expression is strikingly associated with the apoptotic effect of sunitinib and may be a biomarker for sunitinib sensitivity in ccRCC patients.

## CONFLICT OF INTEREST

There are no potential conflicts of interest to disclose.

## AUTHOR CONTRIBUTIONS

ZSW, YLP, and LBX performed the experiments, analyzed the data, and wrote the manuscript. ZLZ, FJZ, and CPY conceived the study, participated in the study design and revised the manuscript. PD provided patients information. JW prepared the human samples and paraffin section. ZL and KN collected the clinical data. MHD, NW, WWS, and ZYL help with the main experiments. All authors approved the final version of the manuscript.

## ETHICAL APPROVAL STATEMENT

This study was with the approval of the Institutional Research Ethics Committee of Sun Yat‐sen University Cancer Center and the Laboratory Animal Ethics Committee of Sun Yat‐sen University Cancer Center.

## Supporting information


Figure S1

Figure S2

Data S3

## Data Availability

Data in this study were available.
