# Supplementary figures and images for: Role of Sam68 in Sunitinib induced renal cell carcinoma apoptosis
Source: Cancer Med. 2022 Apr 10;11(19):3674–86. doi: 10.1002/cam4.4743 (PMC9554455; doi:10.1002/cam4.4743)

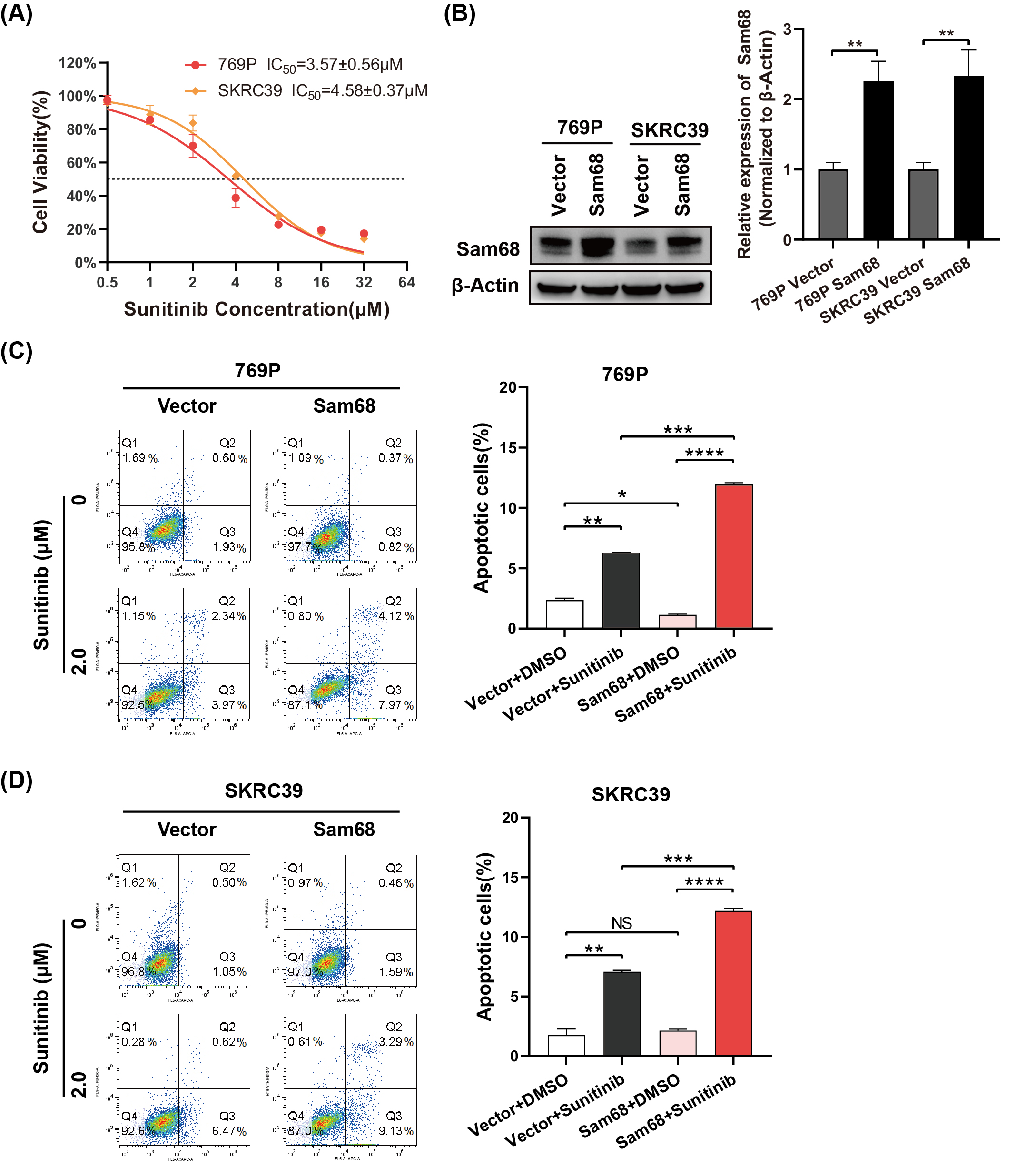

Supplement: Supplementary file 1 — Figure S1 [file CAM4-11-3674-s001.jpg]

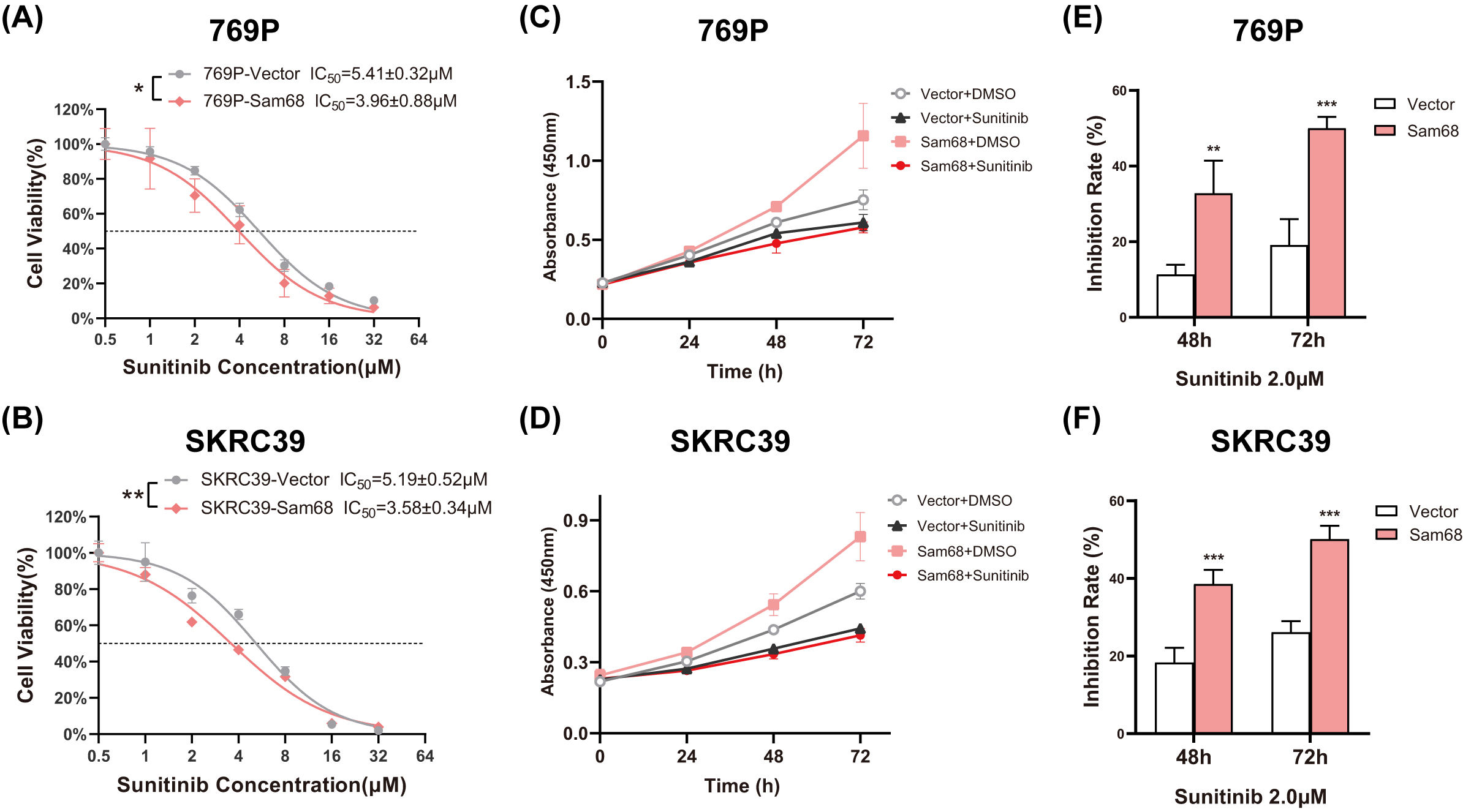

Supplement: Supplementary file 2 — Figure S2 [file CAM4-11-3674-s002.jpg]
