# Supplementary material for: Role of Sam68 in Sunitinib induced renal cell carcinoma apoptosis
Source: Cancer Med. 2022 Apr 10;11(19):3674–86. doi: 10.1002/cam4.4743 (PMC9554455; doi:10.1002/cam4.4743)
Supplement: Supplementary file 3 — Data S3 [file CAM4-11-3674-s003.docx]

**Supplementary figure legends**

**Figure S1. Sam68 increases RCC cell apoptosis induced by sunitinib.** A. The dose-response curves and the corresponding half maximal inhibitory concentrations (IC_50_) of sunitinib in 769P and SKRC39 cells after treatment for 48 hours were shown. B. Western blotting verified the efficiency of Sam68 upregulation in 769P and SKRC39 cells. C-D. Flow cytometry plots showed that the apoptosis rates induced by sunitinib were obviously increased in Sam68 overexpressing 769P and SKRC39 cells compared to the vector control cells (left), and the corresponding proportions of apoptotic cells were shown in the bar graphs (right). NS: no significant; *: *p*＜0.05; **: *p*＜0.01;***: *p*＜0.001; ****: *p*＜0.0001.

**Figure S2. Upregulation of Sam68 strengthens the antitumor effect of sunitinib in vitro.** A-B. The half maximal inhibitory concentrations (IC_50_) of sunitinib were lessened in 769P and SKRC39 cells while Sam68 expressions were upregulated. C-D. The growth curves showed that the growth rates were significantly suppressed in 769P and SKRC39 Sam68 overexpressing cells. E-F. The bar graphs showed that the growth inhibition rates in cells were increased after sunitinib treatment for 48 and 72 hours when Sam68 expressions were upregulated. *: *p*＜0.05; **: *p*＜0.01;***: *p*＜0.001.
